# Inner Experience – Direct Access to Reality: A Complementarist Ontology and Dual Aspect Monism Support a Broader Epistemology

**DOI:** 10.3389/fpsyg.2020.00640

**Published:** 2020-04-23

**Authors:** Harald Walach

**Affiliations:** ^1^Poznan University of Medical Sciences, Poznań, Poland; ^2^Department of Psychology and Psychotherapy, University of Witten/Herdecke, Witten, Germany; ^3^Department of Pediatric Gastroenterology, Health Science Institute, Berlin, Germany

**Keywords:** consciousness, materialism, contemplative science, dual aspect model, complementarity, ontology, epistemology, introspection

## Abstract

Ontology, the ideas we have about the nature of reality, and epistemology, our concepts about how to gain knowledge about the world, are interdependent. Currently, the dominant ontology in science is a materialist model, and associated with it an empiricist epistemology. Historically speaking, there was a more comprehensive notion at the cradle of modern science in the middle ages. Then “experience” meant both inner, or first person, and outer, or third person, experience. With the historical development, experience has come to mean only sense experience of outer reality. This has become associated with the ontology that matter is the most important substance in the universe, everything else—consciousness, mind, values, etc., —being derived thereof or reducible to it. This ontology is insufficient to explain the phenomena we are living with—consciousness, as a precondition of this idea, or anomalous cognitions. These have a robust empirical grounding, although we do not understand them sufficiently. The phenomenology, though, demands some sort of non-local model of the world and one in which consciousness is not derivative of, but coprimary with matter. I propose such a complementarist dual aspect model of consciousness and brain, or mind and matter. This then also entails a different epistemology. For if consciousness is coprimary with matter, then we can also use a deeper exploration of consciousness as happens in contemplative practice to reach an understanding of the deep structure of the world, for instance in mathematical or theoretical intuition, and perhaps also in other areas such as in ethics. This would entail a kind of contemplative science that would also complement our current experiential mode that is exclusively directed to the outside aspect of our world. Such an epistemology might help us with various issues, such as good theoretical and other intuitions.

## Background—Epistemology is Tied to Ontology

Epistemology, our understanding of how we arrive at knowledge about the world, and ontology, our understanding of what the world consists of, are intimately tied together. This is easy to see, and in the introductory part of this essay, I will describe this relationship between ontology and epistemology. If we consider our world to consist of material entities only, then what we need are methods of discovering these entities and making sure we reduce errors and insecurities in describing the nature of these material entities, how they are related, what they consist of, etc., to a minimum. Our scientific model of understanding the world has been very successful if we consider the progress we have made in understanding our natural world around us. This is partly due to the fact that this scientific model has originally restricted itself to understanding the material world. In order to arrive at reliable knowledge, the scientific epistemological model has started from the assumption that the material world consists of material particles only and has restricted itself to discovering the forces that act on these and the laws that govern these forces. This was, by and large, the success of the approach started by Galileo, Descartes, and Newton (Burtt, 1932; Descartes, 1954; Fischer, 2015; Maxwell, 2017). It operated on idealized material particles in mutual exchange of energy and their movements. The issue became more complicated with the advent of quantum physics, but with some rounding and assumptions, one can derive classical physics from quantum physics and can still use the general approach of classical physics to all macrophenomena, from chemistry to biology and neuroscience (Primas, 1981, 1994b).

This fruitful approach of science is based on observation of the external world and theoretical models of how these observations can be accounted for, as every observation is always dependent on a theoretical model we have of our world and the predictions such a model makes. This is known as the “theory-ladenness” of observations (Hanson, 1969/2018). With the progress of science, such models became ever more abstract and complex, making counterintuitive predictions, such as in quantum physics, defining specified realms of observation. Finally, it is always the observations that are the arbiter on the structure of reality. These observations happen originally through our senses and, in scientific observation, using enhancers such as telescopes of various kinds or microscopes, immunological or biochemical assays. All the same, these subtle enhancers are enhancers of our sense perceptions, mostly seeing, and they are directed toward material objects in the world outside. Since the time of Roger Bacon, i.e., since the 13th century, the experiment as an active manipulation of our objects of interest has been added to the epistemological arsenal (Crombie, 1953; Bacon, 1983, Bacon, 1998; Lindberg, 1992, 1997)^1^. While in observation we purely observe, without intervening, natural developments and movement, for instance of stars, or plants and animals, in experimentation, we interfere with their normal dynamics. This is only possible for some of the subject matters of science. We cannot interfere with the movement of stars. But we can interfere with the life cycles of single plants, animals, cells, and meanwhile perhaps even with the evolution of our planet, and we can experiment with chemical substances in biological structures to develop and test pharmaceutical agents, etc. Nevertheless, experimenting is a kind of observation as well, only more directed and purposeful. As Francis Bacon, in the 16th century, said, echoing the tentative steps of his namesake of the 13th century, Roger Bacon (Bacon, 1859, Bacon, 1267/1897): If we direct observation purposefully, we call it experiment, if it happens by accident, we call it experience (Bacon, 1990, I.82).

The ontological assumption behind this is quite clear: What we observe is the material world outside, in contrast to the inner world of our dreams and phantasies. Thus, our observations refer to objects in the world, the nature outside. Therefore, the subject matter of science or the object of interest is nature, the world outside, and its constituents. For a long time, it was quite rational and useful to assume that this is constituted of material objects only or mostly because this is what we can see with our eyes. There might have been invisible things out there as well, such as spirits, ghosts, or suchlike things. But the progress of science and its associated program of ontological reduction has led us to believe that invisible things, if they exist, can always be traced and reduced to material objects or their reverberations (Agazzi (ed.), 1991; Primas, 1991; Dupré and Nicholson, 2018). Electromagnetic radiation would be a pertinent example. We cannot see electromagnetic radiation, except within the small window of visible light. If the wavelength is too long, there is only a small window where we can feel it as infrared radiation or warmth. If the wavelength is too short, we cannot even feel it, let alone see it. We, at most, feel the distant effects, when we suffer from sunburns, or even more distant effects such as genetic deviations that manifest as skin cancer. Other types of electromagnetic radiations, for instance the microwaves from mobile phones, we do neither see nor feel. But we do have a good theoretical model for these kinds of radiation, and this theory tells us that it is carried by minute particles, photons, that can be identified, even though they do not have a mass, by their effects. Their vibration frequency can be measured and various other aspects. Thus, we have succeeded in reducing invisible radiation to material entities. In the same vein, natural science starts from the assumption that everything that is there, worthwhile to discover, is in fact material in essence and hence can be seen, heard, felt, or smelled. Thus, the epistemological mode of science—to discover what is there in the world—is tied to its ontological assumption, which currently is: what is there to discover is in principle material in kind, or, if it is not material prima vista, it can be reduced to material entities.

It might be worthwhile to remind us of the fact that other ontologies lead to different epistemologies. If a society or culture believes, for instance, in the reality and importance of spiritual entities, it normally also develops methods and modes to experience them or, the other way around, develops ontologies in accord with their experiences. Indian shamans, for instance, believe that there are spiritual guides and plant spirits that can be contacted, and have devised rituals to make them experientially available or arrange for their services, for instance in the Ayahuasca ritual (Krippner and Sulla, 2000; Shanon, 2002; Ferrer, 2013, 2018). This is meant to contact plant spirits and other spirit guides to learn about potential healing strategies or other important things. Now, clearly, from our Western scientific point of view, these experiences are considered hallucinatory or imaginary, as we think that there is no outside referent here that can be addressed as a “spirit.” This is so because we start implicitly from the assumption that it is “all in the brain,” or more precisely, we implicitly start from a concept where mental phenomena and experiences are dependent on the brain, and they either have an outside referent, such as in sense experience, or else they are imaginary, as in dreams, or Ayahuasca experiences, for that matter.

Another example how a different ontology leads to a different epistemology is Indian Vedanta or Hindu psychology (Akhilananda, 1960; Rao, 2005; Fulton, 2008; MacPhail, 2013, 2017; Sedlmeier and Kunchapudi, 2016). Here, the ontological assumption is that the ultimate reality is spiritual in nature, consciousness or spirit, Brahman, the ultimate spirit, and Atman, the individual spirit of our individual, higher consciousness that is part of Brahman or an expression of Brahman. That is the reason why the major striving in this culture is the purification of consciousness and the emphasis on inner experiences during meditation or higher states of consciousness, such as in the state called Samadhi. This is a state in which individual consciousness has emptied itself from cognitive content, such as categorical thinking, emotions, imaging, and is dwelling in pure presence. Vedanta psychology knows various states of this kind, but we leave that aside for now. The important thing here is: in such a state of Samadhi, which can be broadened in time and scope and can become a second nature, the individual consciousness is thought to reside in a general unity with the larger consciousness and can discover important aspects of it. That is likely the reason why Indian culture, over the centuries, and perhaps Asian culture in general, as a rule, has emphasized more the inner world, while the Western mind was more bent on discovering the laws of the material world and paid comparatively little attention to the workings of consciousness (Green, 2016). Ontology drives epistemology, and epistemology implicitly strengthens or explicitly produces ontology. The two aspects about our belief of the world are interdependent. What we think the world is made of will determine how we try to understand it and learn about it, and the primary mode of our relating to the world will drive how we see it and what we think is extant in it.

## The Traditional Materialist Stance of Science Allows Only an Empiricist Model of Outer Experience

Our traditional Western science, we already observed, is mainly directed toward the outside world. This has become a more and more materialistic enterprise, relying mainly on an empiricist model of epistemology trying to unravel those material entities in the outside world and their relationships. Thus, ontology reinforces epistemology, which in turn reinforces the underlying ontology. It will be useful to survey in this paragraph the history and systematic relationship of these two anchors of the modern scientific stance: materialist ontology and empiricist methodology.

At the beginning of University scholarship, in the 13th and 14th century, it was pretty clear that there are both material and spiritual entities in the world. Therefore, two different modes of experience were necessary, one directed to the outside world, experience as we know it, to learn about material entities, and one directed toward the inner world of experience to learn about the spiritual world, what we would today call first-person experience or inner experience. This has changed since. One important watershed was Roger Bacon’s Opus Majus, his sketch how he would envisage a future universal scholarly learning and university model (Bacon, 1267/1897; Power, 2012). He wrote this book at the request of the Pope, whom he knew personally from his previous position as papal legate for his home country, England, and sent it to him in 1267. In it he described how he envisaged a future universal scholarship. Such future scholarship was based, he held, on experience in general and employed mathematics and good knowledge of language, Latin of course as the lingua franca of Western scholarship, but also Hebrew and Greek. Importantly, he saw experience as bimodal^2^. One part of experience, he said, was outward directed, toward the world and the senses and was the source of our general—philosophical, he said—knowledge of the world. The other part was an inner type of experience or spiritual experience. This he identified with the mystical path of inner purification and mystical union, which he had taken over from an earlier writer, Thomas Gallus, who had put together the Franciscan mystical path in a small book (Thomas Gallus, 1243/1503). It was the mode how our patriarchs, prophets, and forefathers had gained knowledge. This, he felt, is necessary to make sense of the other types of experience. It was also necessary for practical purposes of “guidance and political decisions.” Bacon’s Opus Majus existed only in three exemplars. One went to Rome and was buried in the Vatican library. The Pope had received it, but he probably never read it because he died shortly after having received the text. But Bacon’s text was copied from his own copy by some of his friar brethren of the Franciscan study house in Paris, where Bacon wrote it, and so made its way into the world. The Vatican copy was eventually discovered by Pico della Mirandola and made its way out into the world, where the younger Bacon, Francis Bacon, eventually got his ideas from Mandonnet (1910); Newbold (1921). A lot of what Francis Bacon, who was no relative of the older Bacon, teaches—all his teachings about the idols—he found in the older Bacon’s text, and a lot more, for instance the emphasis on experience and experiments.

But what had been lost for good until recently was the older Bacon’s emphasis on the two-pronged approach of experience, one inner and one outer mode. What was kept and nourished was “outer” experience or sense experience. This became the dominant mode of Western science, refined, aided by multiple tools and skills. But it was only half of Bacon’s notion of experience, nevertheless. This was probably a reflection of the rise of an implicit world model that relegated all non-material entities to domains other than science—to private philosophy and mysticism, religion, literature, poetry, and art. By the end of the 17th century, Descartes had paved the way for a separation of cultures. The culture of science was directed toward the outer, material world. The culture of philosophy and religion dealt with the mind, faith, beliefs, and invisible entities like mathematical truths. In the end, after the philosophers of the enlightenment movement had moved science forward as the modern enterprise that would set human minds free from ideological, dogmatic, and political bondage (Dupré, 2004), science and its epistemological mode had only the material world as its object. All other kinds of knowledge acquisition, philosophical reasoning or insight, were no longer considered scientific. After Kant philosophy tried to exercise its critical function, criticizing the cultural dominance of natural science, for instance when Husserl and later Heidegger wrote about the scientistic deviation of natural science, and in their wake, French constructivists and deconstructionists pointed out the social, political, and economic preconceptions that are always present (Heidegger, 1967; Husserl, 1909/1977; Foucault, 1974/1991).

From there, an important tradition arose that took up the strand of inner experience and thus also a different type of ontology, the tradition of phenomenology. It took its origin in the philosophy of Franz Brentano, who had set out explicitly to reform philosophy (Tiefensee, 1998; Binder, 2019). His idea of reform was to adapt the method of science, experience. This, for Brentano, was a systematic way of introspection or inner experience, using the terminology adapted here. Brentano’s most famous student was Edmund Husserl, who inspired the French tradition of phenomenology (Merleau-Ponty, 1966; Zahavi, 2003; Husserl, 1930/2009). This was, eventually, also connected back to neuroscience: Varela was the first to postulate a phenomenology of experience as a complement to neuroscientific methods (Varela et al., 1991). This has inspired a strand of research within neuroscience that calls itself contemplative neuroscience (Shear, 2007; Berkovich-Ohana et al., 2013; Dor-Ziderman et al., 2013; Jo et al., 2013, 2014, 2015; Lutz et al., 2015; Winter et al., 2020). This refers to a scientific model of experience, where neuroscientific methods, such as electroencephalogram (EEG), MRI, magnetoencephalogram (MEG), or others, are used to understand brain states or dynamics—experience of the outer world or third-person types of experience. As a complement, inner phenomenological accounts of the subjective experiences of research volunteers are collected and put into relationship with these brain states. Also inspired by the phenomenology of Husserl and Merleau-Ponty are modern revivals of systematic introspection (Petitmengin and Bitbol, 2009; Bitbol and Petitmengin, 2013). They are being used to understand experience directly, for instance as forerunners of epileptic fits (Petitmengin et al., 2007; Heinen, 2012) or as the direct experiential content of reality as such (Petitmengin, 2006, 2007; Petitmengin and Bitbol, 2009; Weger and Wagemann, 2015). This phenomenological tradition is a very important and rich new movement. It adopts a different epistemology and hence also leads to a different ontology (Varela, 1981a,b, 1984; Ferrer, 2018): There is no split between mental and physical phenomena because each statement about the outside world starts from lived, embodied experience. This, indirectly, illustrates my point: Epistemology and ontology are tied together. However, even phenomenology was unable to break the stride of current science toward rampant materialism, although it added important nuances and caveats.

What had been lost along the way was a viable concept of “mind” or “consciousness” that would be ontologically anchored and thereby support its own epistemological mode (Beckermann, 1989). Descartes’ dualism was philosophically problematic, as philosophers in his immediate temporal vicinity, such as Spinoza and Leibniz, had pointed out. But it was the starting point for exorcizing, ostracizing even, all things mental from the remit of natural science. The program of reduction of all biological things to machines and mechanical workings that Descartes had started with his Traité de l’Homme published posthumously in 1664 (Descartes, 1664/2003) surreptitiously also moved over to the mind when scientists started to consider the brain as the organ that produced the mind or, more radically even, as identical with the mind (Armstrong, 1968; Churchland, 1986, 1988). Even more radical, in modern times, some scientists considered the mind superfluous as an entity and thought it sufficient to understand the workings of the brain (Dennett, 1991). Never mind that this program of neuroreductionism, i.e., the assumption that a thorough neurological understanding of the processes in the brain would give us a full understanding of the workings of the mind, has not produced a satisfactory theory as yet and very likely will not do so for some time to come, and perhaps never (Hasler, 2015). The abstraction that only material things are really important is still very powerful, or put differently, the historical consequence of the process set into motion by Descartes, Newton, the enlightenment philosophers of France and England, and the historical success of the natural sciences has led to an implicit materialist ontology, not only of science itself but also of our whole scientific culture at large. This has spilled over into our popular culture, where rampant materialism in the way we are treating our planet and in the dominance of the capitalist economic model has become the mainstream.

This ontological model of materialism, even though only implicit in many fields, supports, consequently, the epistemological model of empiricism understood as the idea that we only need sense perception to learn about the world. Such scientists Baas van Fraassen has called “naturalistic natives” (van Fraassen, 2002, 2016) because they cannot even imagine that this is a very limited, flawed conception. Yet, it has become the dominant mode (Principe, 2016; Robinson, 2016; Williams and Robinson, 2016). It is associated, ontologically, with the idea that consciousness, or mind, is secondary to, dependent on, derived from, brain activity, or as stated above, only brain activity is necessary to understand the mind.

## Conundrums, Paradoxes, and Empirical Anomalies of the Current Model

This stance, however, leads to some conundrums and paradoxes, and some empirical anomalies militate against it. These empirical anomalies and theoretical paradoxes will be explained and discussed in this paragraph.

The consequence of these anomalies and paradoxes, if taken seriously, is that we very likely need to be open to the possibility that another mode of relating to the world, or a broader kind of epistemology, will be needed. This will be one, where inner experience, very much along the lines as envisaged by Roger Bacon, is needed to complement our sense experience. It will be an additional mode of insight, and it is dependent on and derivative of a different ontology. This broader ontology leaves room for consciousness or mind as a coprimary entity to matter in a complementarist model of mind–body relationship or consciousness–matter duality that is no longer reducible to either matter alone, as in a materialist monist model, or mind/consciousness alone, as in a traditional idealistic perspective. The most parsimonious and probably theoretically most honest approach is to say that we need a model, in which neither matter is reduced to mind nor mind to matter, but where both are coprimary or perhaps phenomenologically secondary to a primary reality that we cannot experience otherwise than in its dual aspects of mind/consciousness and matter. Before I work this out and develop the consequences for epistemology, let me give a few arguments why I think this is necessary.

### Abduction or Creative Insight

The empiricist stance that believes that sense experience and thinking about those sense experiences, together with some abstract reasoning and mathematical modeling are sufficient does not really work if we look carefully. This stance overlooks an important ingredient in the scientific process. Epistemologically speaking, the well-known steps of induction—observing single instances of empirical occurrences and then deriving some general statement from it—and deduction—using such a general statement and then deriving sentences from it that can be empirically tested or are known to be true because they follow strict logical deductions—are not sufficient to found and support science. Although they are the most important, most frequently employed and best-known modes of scientific activity, they lack an important step that C. S. Peirce has pointed out. But it can already be found in the oldest epistemological model in the West, in Aristotle’s organon (Aristoteles, 1990). Peirce called it abduction. His definition for it was “facts in search of a theory” (Peirce, 1931, VII, 218). By that, he meant that we normally start not from brute, simple facts but immediately put facts and empirical findings together into models. Aristotle called this process “anchinoia,” which was translated by one of the first Latin translations and commentaries into “sollertia,” meaning “sharp-sightedness” (Grosseteste, 1981, I.19, p. 281 ff.). Aristotle pointed out that behind this process was what he called “insight—noesis.” Insight refers to the capacity of the human mind to “see” relationships or derive unseen relationships behind facts. It is, as it were, the theorizing capacity of the human mind. Now, the question is: how does the mind arrive at these theoretical structures, or, put differently, how do we arrive at meaningful theories that we need to organize our perceptual reality in the first place and to do useful science in the second place? Einstein used to say: “Ideas come from God” (Brian, 1996, p. 61). By that, he did certainly not mean any personal transcendent entity, as Einstein was an agnostic, but he meant that ideas had a different status from observations and empirical facts. They cannot come from observations. They are needed to understand, collect, and order observations. Hanson used the expression that observation is “theory-laden” to describe this fact (Hanson, 1969/2018). But how do we arrive at theories? Most of us use the theories that we are taught and find when we start with the business of science. But those theories have been arrived at by some highly creative and inspired thinkers in the first place, by Newton, Descartes, Einstein, Heisenberg, Planck, and Schrödinger, to name but the more prominent ones. In each and every case, one can locate biographically a certain insight, a creative idea, a sudden “inner” theoretical experience, before a formal theory was developed. Where did it come from? Was this pure accident? It very likely was a mix of careful study, long attempts at problem solving, a huge amount of background knowledge, and at some point a specific inner experience that helped bring all the details together such that a meaningful theoretical structure could emerge. This is the process that Peirce called “abduction” (Fann, 1970). It is interesting to observe, just as an aside, that we have managed to build extremely powerful computers that are highly efficient in performing deductive and inductive processes. But to my knowledge, we have not succeeded in building a computer that can perform abductive steps reliably (Penrose, 1990; Collins, 2018)^3^. Perhaps, they will one day, but until it is done, it seems safe to state that this type of reasoning, or insight, is a human prerogative.

Thus, the conundrum or paradox here is: The materialist stance assumes that all mental operations are physical and material in nature, namely neuronal activities in the brain. Yet, it presupposes a conscious mind to state this in the first place, a lived experience in the language of phenomenology. It needs a specific mental process to find fruitful scientific theories, and this process seems to be not algorithmically reproducible. It has a lot of phenomenological similarity with the type of experience I call “inner experience.”

### Consciousness Unexplained

As a secondary paradox, one can prolong this thought: Only once the materialist stance to explain all mental activity, all insight, all qualitative experiences has been fully worked out in a theory of ontological materialist reductionism can the epistemological stance of pure empiricism become a full-fledged dominant program. But we do not have such a theory. What we have are promises and stipulations (Nagel, 2012). But good arguments have shown that these promises are likely hollow and the stipulations wrong. Chalmers has shown that a supervenience theory of consciousness does not work (Chalmers, 1996, 2010). Hoche has pointed out that an identity theory of mind and matter makes a severe category mistake (Hoche, 2008). Arguments like those advanced by Jackson, Nagel, Maxwell, Noë, Velmans, and others point to the fact that the qualitative aspects of consciousness will not be and cannot be captured, in principle, by materialist approaches because a materialist theory can only describe the outer workings of neuronal activities (Maxwell, 1968, 2000, 2011; Nagel, 1974, 2012; Jackson, 1986; Velmans, 2007, Velmans, 1993/2007, 2009; Noë, 2009). Even if we know everything about another person’s brain, we do still not know what it feels like, for this person, to sense pain, experience bliss, or taste aged Pinot Noir. Already Leibniz had pointed out in his famous “mill parable,” revived in recent times by Bieri, that if we imagine our brain to be like a mill such that we could walk into it and inspect it, we would only find mechanical activity, but no thoughts, emotion, imaginations, desires, and the like (Leibniz, 1966, §17; Bieri, 1995).

The fact that neuronal networks have succeeded in imitating some feats of human cognitive systems, like pattern recognition, some aspects of learning and complex interactions, only shows that certain aspects of human, and for that matter animal cognition, can be implemented in technical systems. But as Searle’s Chinese room argument has shown, this does not prove anything about consciousness and understanding (Searle, 1992). If it is complexity that drives consciousness and if consciousness is an emergent property of a large assembly of neurons or similar kind of relays, why is the immune system not conscious, or our cerebellum, or the internet, or the bacteria in our guts that outnumber our body cells by one order of magnitude and that are well connected, let alone all the other bacterial cultures that outnumber both us humans and our neurons? They are also extremely well connected and perform feats like swapping resistance genes between different strands and behaving cooperatively, apparently and obviously all without consciousness, culture, and language.

I am shortening a lively discussion that is currently carried out in numerous specialized publications and at the “The Science of Consciousness Conferences.” It is fair to say that a growing consensus of the community of consciousness researchers is unhappy with the current implicit mainstream consensus that a materialist model of the world and the brain is sufficient to understand consciousness. There certainly is a very strong correlation between brain and consciousness. Nobody is really denying that. But how the two relate is unclear. At best, a dual aspect monism seems to be viable, in the sense that consciousness is coprimary with matter as has been propagated by Spinoza and his followers (Spinoza, 1977; Jonas, 1980; Epperson, 2009).

### Anomalous Cognition and the Parapsychological Database

We and others have argued that the database of parapsychology challenges a materialist–reductionist view of consciousness, as well (Costa de Beauregard, 1998; Walach and Schmidt, 2005; Kelly et al., 2007, 2015; Beauregard, 2014; Cardeña, 2014, 2015; Baruss and Mossbridge, 2017; Radin, 2018; Schwartz et al., 2018; Walach, 2019). Or, turned around, the fact that a materialist ontology is still the implicit mainstream model in science is likely the reason why the large and consistent database of parapsychology is not taken seriously as it would deserve (Braude, 1987; Cardeña, 2015, 2018; Reber and Alcock, 2020). Meta-analyses that span decades of carefully controlled experimental research have documented that telepathy, extrasensory perception like remote viewing, precognition, and presentiment, probably also micropsychokinesis are factual and statistically robust phenomena (Schmidt et al., 2004; Storm et al., 2010, 2012, 2013, 2017; Mossbridge et al., 2012; Schmidt, 2012; Bem et al., 2015; Walach et al., 2015; Storm and Tressoldi, 2017; Cardeña, 2018; Duggan and Tressoldi, 2018; May et al., 2018). They are not in the same sense causally available as light switches are because we do not understand these phenomena well enough and perhaps because they cannot be engineered at will in the same sense as standard material-causal phenomena. But nevertheless, they are robust, and standard explanations like fraud, publication bias, and perceptual errors are no likely explanations. Already William James, in his presidential address as president of the Society of Psychical Research in 1896, used the fagot argument that applies here (James, 1896): In a fagot, each single stick can be broken easily, but the fagot as a whole cannot be broken. In the same sense, the evidence of parapsychological phenomena such as clairvoyance, telepathy, remote viewing, precognition, and psychokinesis can be dismantled study by study, if one is bent on it, or single problems might be identified with single pieces of research, but the evidence as a whole is difficult to dismiss. What was true for William James at the end of the 19th century is even truer for today’s database after 40 years of diligent research.

The overarching feature, we and others have pointed to, is the inherent non-locality of psychic phenomena. Precognition defies the locality condition of special relativity. Telepathy is a decidedly non-local phenomenon because neither time nor distance seems to play a decisive role (Carr, 2015; May and Marwaha, 2018; May et al., 2018). Those who are able to use telepathy are not bound, it seems, by distance or time (Targ and Puthoff, 1974; Targ et al., 1979).

Why is this relevant to our discussion of ontology and epistemology? The relevance comes from three interrelated facts.

For one, the current implicit mainstream materialist ontology operates on a notion of causality that is purely material in nature. Wherever causes transmit effects, material particles—photons or other exchange particles, molecules, and the like, shorthand for “signal”—mediate such effects. These signals are bound by the framework of the special theory of relativity to operate at the limit of the speed of light. This means that there are only causes that can travel at the speed of light. This is what is often referred to as the “locality principle” or the “local” nature of causes. It means that causes are in nature material. This is also the reason why the idea of a non-material influence on material systems is alien to this type of thinking. For that reason, a (non-material) conscious will influencing the brain is just about as strange as a non-material intention influencing a distant material system without any mediating cause.

Second, within the underlying materialist ontology, it is very difficult to see how consciousness, as secondary to material events in the brain and derivative thereof, can have a direct, unmediated effect on other minds, as in telepathy, or on distant material systems, such as in psychokinesis. In a classical standard materialist ontology, such effects would necessitate—material—exchange particles to mediate such effects. But within the current world model, there is no room for extra particles to mediate such types of effects. Hence, we would either have to change our world model, which no one really wants to do, or we have to agree that there is an element of direct “influence” between conscious systems and other conscious or material systems, without the mediating effect of material exchange particles. This is what technically is referred to as the inherent “non-locality” of effects of anomalous cognition and action, such as telepathy, clairvoyance, and psychokinesis.

Finally, precognition effects are direct violations of the current model of locality and material causes, as they contradict the core tenet of special relativity that all causes have to travel at the speed of light, and hence, as a direct consequence, the future is open and cannot transmit information about its state. Exactly this is what is experienced in precognition and violated in some kinds of experiments.

Thus, these features of anomalous cognition and action challenge directly the fundamental tenets of an implicit mainstream materialist ontology, a fact that has been clearly observed and voiced by critics recently (Reber and Alcock, 2020). By challenging the materialist ontology, the anomalous empirical observations discussed above imply a broader and less restrictive ontology, in which consciousness is coprimary with matter, as we will see shortly. Using such a broader ontology will also give space and reason to apply a broader epistemology.

Critics try to eschew this empirical challenge by pointing to the difficulty of replicating such findings (Alcock, 2003). It is certainly true that the findings of parapsychology are difficult to replicate, and within our own model, such a problem is even predicted for theoretical reasons (Lucadou et al., 2007). But it should not be overlooked: replication is a general problem in psychology, where less than 50% of standard experiments turned out to be replicable (Open Science Collaboration, 2015) or, for that matter, in medicine, where the difficulty to replicate is a genuine problem even for well-understood mechanistic interventions (Ioannidis, 2005). Thus, the difficulty to replicate is generic across all sciences that deal with human beings. It is difficult to defend a strategy of demanding better replicability from the vantage point of a science that has not solved this problem in the first place, I feel. Apart from that, granted the fact that direct replications are difficult in anomalous cognition research, across the board, overall and considering all experimental paradigms, the strong effects of meta-analyses that cannot be accounted for by publication bias or fraud need to be taken seriously (Cardeña, 2015, 2018).

Often, critics voice the argument that, if psychic phenomena were true, they would contradict our “laws of nature” (Reber and Alcock, 2020). This is only true if by “laws of nature,” we in fact mean a Newtonian mechanistic world view. But in a world where science is unclear about the whereabouts of about 95% of all matter and energy, where the most widely accepted theories, relativity theory and quantum theory, are at odds with each other, it is not very difficult to imagine a different type of physics that will have no problem integrating such phenomena. We have shown, for instance, that a generalized version of quantum theory that is a systemic model and not restricted to physical and material systems can be used to theoretically understand and reconstruct psychic phenomena (Walach et al., 2014). Others use different physical models, for instance hyperspatial models, that would be able to account for such phenomena (Carr, 2015). These examples should suffice to state the obvious: Psychic phenomena are empirical phenomena that are at odds only with a localist, materialist world view. As soon as such a stance is abandoned and phenomena trump theories or, rather, dogma, then they become quite natural.

As such, they challenge the materialist world view and the associated epistemology and demand a broadening. A minimum consensus model we propose is a dual-aspect monist model or, to use a more precise formulation, a complementarist model. This would also generate a different kind, a broader epistemology, to which we now turn.

## A Broader Ontology and Epistemology And, Finally, Methodology

If the arguments above are accepted, at least tentatively, to see where they lead to, then we would need a broadening of our ontology. Matter alone, it seems, is not sufficient to account for the phenomena we experience, beginning with our consciousness and leading to, but probably not ending with, psychic phenomena. Thus, we would have to stipulate that consciousness is at least as primary as matter. Therefore, we call it coprimary. This means that neither is matter primary, and consciousness derived from it, as a kind of emergent property, or as a secondary aspect of material organization nor is consciousness or mind primary, as in classical idealistic models, where matter is secondary to mind or consciousness. Both monisms, materialistic and the idealistic monisms, have a similar problem, namely, making plausible how a categorically completely different entity, matter, or consciousness can be derived from the original substance. They can only make this happen by allowing only a subsidiary ontological status to the other entity. During times when idealistic thinking was rampant, matter was denigrated and marginalized and with this process science, the study of the natural world. During our times when materialistic thinking is the dominant mode, the opposite happens. Consciousness and related phenomena are marginalized and whatever is associated with it.

Neither model is satisfactory. A model in which matter and consciousness are coprimary I will call complementarist, which I find a stronger version of a dual-aspect model (Walach and Römer, 2000, Römer, 2012; Walach, 2005; Römer and Walach, 2011). The prototypical dual-aspect model is Spinoza’s. Here, a unitary substance that is not defined shows itself in two ways, as mind and matter. But their relationship is not clarified further. The same can be said for modern dual aspect monisms like that of Max Velmans (Velmans, 1993/2007, 2009), to give a prominent example. The ontological or shall we say phenomenological difference between the two aspects is emphasized. But there is no further qualification, except that they are both necessary for describing a human being. The complementarist stance that we will advocate here describes these two phenomenological perspectives as complementary in the sense that Bohr introduced the term into physics (Bohr, 1966). He used it to describe the paradoxical behavior of quanta that can be, in one experimental setting, seen as particles and in another as waves, but never as both. Yet, both descriptions are necessary to define the particle in full. Thus, complementary perspectives are perspectives in which we need two mutually exclusive descriptions that unite incompatible perspectives into one and the same phenomenon to describe a complex entity, such as a conscious human being. Already Bohr had uttered the suspicion that complementarity as an epistemological concept might be useful to understand the mind–body duality (Jordan, 1947, Jordan, 1980; Meyer-Abich, 1965; Brody and Oppenheim, 1969; Fahrenberg, 1992; Röhrle, 2001).

In his exchange of letters and ideas that lasted from 1932 until his death in 1958, Wolfgang Pauli, one of the sharpest minds of the physics community of his time and one of the founding fathers of quantum theory, suggested to Carl Gustav Jung, the Swiss psychiatrist, that physics would only be complete as and when psyche or consciousness was integrated into physical theories (Meier (ed.), 1992, 2001; Atmanspacher and Primas, 2006). He also used the concept of complementarity, at least implicitly, when he, for instance, agreed to Jung’s suggestion to see causality and synchronicity in the same sense complementary as energy and four-dimensional space–time (Meier (ed.), 1992, p. 64). Implicitly, both followed along the same trajectory. Jung had suggested seeing psyche or mind, and matter, as two aspects of an underlying unity, which he called, drawing on the Neo-Platonist tradition of the alchemist literature, “unus mundus,” “one world.” By that, he had in mind a kind of transcendent, ultimate reality that we humans do not have access to.

**FIGURE 1:**
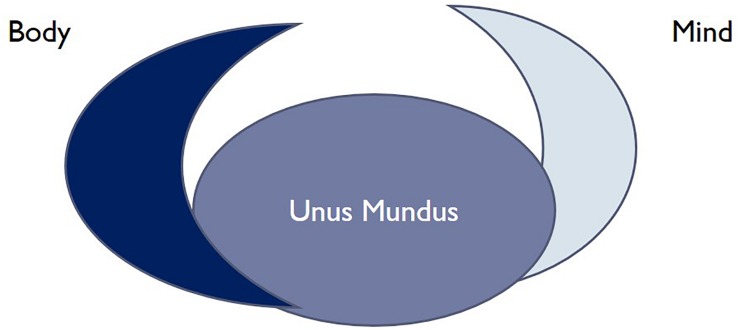
A representation of a complementarist model inspired by Jung’s idea of unus mundus as underlying psyche and matter.

Using the quantum-mechanically inspired thinking of a modern interpreter of Jung, the late Hans Primas, we might say that the Jungian “unus mundus” is the endo-physics, or endo-world, of the unitary development of the deterministic Schrödinger equation, where everything has a timeless presence and is non-locally correlated with everything else. Sometimes, Primas also likened this to the Platonic world of ideas or mathematical truths (Primas, 1994a, 1996, 1997. By that, Primas referred to the fact that there are two quite opposing views of interpreting quantum physics: The Schrödinger equation in fact describes a deterministic system of pure possibilities. This is what Primas and others call “endo-physics,” the internal, but inaccessible state of the world, similar to the realm of Platonic ideas. But what we experience is the clear unfolding of definitive events. Here, we see no possibility waves, as in the Schrödinger equation, but definitive chairs and tables. Here, facts are delineated and identifiable. But how the potential of the Schrödinger equation collapses into the actuality of facts is unclear and a matter of dispute. From the point of quantum mechanics, the big riddle is: How do we actually arrive at the classical world we live in and we know (Römer, 2012)? The current generally accepted interpretation is that this is a random process. We can use this distinction between endo- and exophysics to understand the difference between the underlying reality, unus mundus in Jung’s terminology, and our phenomenological world. The endoperspective is normally not available to us humans. Sometimes, in specific moments, we might have temporary and fleeting experiential access to it. Those might be moments of a specially gifted insight, of deep relatedness with someone else, etc. But normally, this realm is beyond our ken. But it appears to us in two seemingly mutually exclusive perspectives, as matter and mind, or consciousness, and in our own existence, we combine these mutually exclusive perspectives: as a conscious human being. I have tried a graphical depiction of this model in Figure 1.

Adopting this model, we see immediately an epistemological consequence. Epistemologically, we now have two potential perspectives for knowing the world. We have our senses and sense experience to experience reality from the outside: outer experience, as the dominant mode of science. But we now also have a second mode, inner experience, the mode of contemplative, meditative, or inner experience. This is the mode of serendipitous insight (Peirce), of inspiration that “comes from God” (Einstein), the mode of insight and sharp-sighted “anchinoia” and “noesis” (Aristotle) that Plato had epitomized in his sixth letter as an insight that strikes one “suddenly,” out of the blue, once one has prepared properly (Plato, 1967, 6th Letter, 341 c 6). Or the mode of contemplative, mystical insight that Roger Bacon drew on and that mystics of later generations, Meister Eckhart, Seuse, and others, spoke of. Perhaps, it is also the direct experience of reality pragmatic epistemologies like those of Zen or other Mahayana-Buddhist approaches refer to when they speak of access to the “dharmakaya,” the real nature of things, or “big mind” (Suzuki, 1970; Hakuin., 1994; Bankart et al., 2003; Dockett and North-Schulte, 2003).

It goes without saying that there are both different gradings and different types of direct intellectual and experiential access to reality. It would be the task of a future epistemology of inner experience to disentangle what I have thrown into one basket. Currently, it is only important to understand: As soon as we adopt such a complementarist ontology, we have, as a rational option of epistemology, also a different access route to reality at our disposal, namely, inner or first-person experience, that might touch reality directly. Exactly how this is going to happen is another question. Perhaps, the phenomenological intuition described by Husserl and Varela is such a mode (Varela, 1996; Husserl, 1930/2009). Perhaps, this is very similar to the bracketing of concepts and the intuitive knowledge aspired to by Buddhist meditators. Perhaps, this is similar to the knowledge medieval mystics had in mind (Walach, 2009, 2010). Perhaps, each mode is completely different, reaches different aspects of reality, and uses different methods. But important for our discussion here is: there is this *principal* option of reaching reality via the route of inner experience. Or put differently: this ontology allows for a touching of reality through inner experience, in addition to the experience of our senses that touch the material reality of our world.

Why would we want to touch on this mode of experience in the realm and within the remit of science, and not relegate this to religion, or spirituality and esotericism (King, 2014)? I suggest it is even necessary to include this mode of insight within the remit of science. For one, a broadened ontology even necessitates such a broader epistemology, and I have given some reasons why we need a broader ontology. Second, only the dispassionately critical and collective searching mode of the scientific culture can make sure that what insights are gleaned from it do not end up in dogmatic encrustations that bolster sectarian teachings and inhuman practices. This is why I have argued in other places for integrating spirituality into the remit of science, which would necessarily lead to a spiritualization of science as well (Walach, 2015, 2017).

This proposal raises a host of questions, though. Perhaps, spiritual practice, such as meditation, just teaches us another experience of the world and not a completely different access to the inner structure of the world? If it does, though, how would we differentiate these different routes? What would be our criteria for disentangling them? What would be our criteria for truth? These are of course complicated questions, which cannot be answered within a paper of limited scope, and they will have to be guiding questions of a future contemplative type of science. Some ideas have already been proposed: The consistency of individual experiences with traditional accounts, the usefulness of such experiences in practices of psychological growth and freedom of individuals, the nourishing of community and connectedness, to name but a few (Walach and Runehov, 2010; Ferrer, 2018). But it is clearly a task for the future to spell out differentiations and criteria of truth, as well as models of practice.

The power of our current empiricist model derives from the seemingly simple methodology of experimentation that is at the core of modern science. This methodological stance connects ontology with epistemology and makes both fruitful. There is no such a pivotal methodological hinge for the envisaged contemplative type of science of first-person experience. After all, it took 500 years to solidify the experimental method. However, introducing a systematic, well-trained introspection, such as in contemplative or meditative practice, directly into the scientific practice and training for that matter might lead to the emergence of such a method, not within the remit of traditional spiritual or religious practices but within the remit of science. As we have no such methodology, it is going to be a project for the future.

Taking the approach I have sketched seriously for a moment, at least tentatively, we see that this ontology allows for a different access route to reality. It offers an avenue to the underlying reality beyond the duality of matter and consciousness. I hold that those empirical and biographical instances that we know of, where gifted scientist came up with theoretical models that render at least part of reality truthfully in highly abstract models, were instances of individual consciousness or mental activity diving into the unity of reality and gaining insights about its structures. Examples are physical theories like relativity theory or quantum theory, biological models like evolutionary theory, or Barbara McClintock who discovered jumping genes by “becoming one” with her plants (Comfort, 2001; Keller, 1983/2003), or mathematical insights like those of Ramanujan and others (Hardy, 1937; Kanigel, 1991). It might be possible to glean even more such insights about deep reality using this mode more conspicuously and systematically. This might speed up our gaining knowledge by avoiding bypasses and detours. But more importantly, it might help us avoid barren roads and dead-end lanes by intuitively avoiding them. Following the Zen stance for a moment, we could surmise that such a contemplative access to reality transcends the seemingly paradoxical structure of the phenomenal world and arrives at the deeper unity that in the Zen tradition is termed “dharmakaya,” sometimes translated to “One Mind.” Insights into that domain might help to fertilize scientific thinking into what Nicholas Maxwell has called “wisdom inquiry” (Maxwell, 1984, 2017). He has been adamant about the fact that seeking knowledge is not enough for science and humanity. Instead, we need knowledge that is useful and will help humanity flourish. But how to arrive at such a knowledge? Will a rational discourse be enough? Can it be rationally willed? Or implemented by managerial decree or by a Vice Chancellor’s orders? I doubt it. Contemplative practice, however, might be a road toward such wisdom inquiry. It might help uncover such deeper lying realities or structures that bring order into hierarchies of potential aims. In other words, it might help to unravel those values that are needed to make sense of and prioritize types and kinds of knowledge. Values, goals, and aims might also belong to the deeper structure of reality. They cannot be discovered in the outside world. They are not available to our senses. And political discourse is only a means of arriving at a consensus or at least at a majority vote about what values we want to prioritize. The current thinking about values is that they are derived somehow from the evolutionary process at large and then negotiated politically (Nagel, 2012; Abele and Wojciszke, 2014; Hands, 2015). But what about the idea that values might be deep structures of reality to be discovered by contemplative practice?

Let us use a more familiar example to illustrate the point here. Everyone is familiar with the sudden discovery of purpose or meaning in one’s life. Normally, this is a personal experience that happens to us. Very rarely do we “make sense,” we find it (Frankl, 1964). When we perform an activity we like, such as pursuing a hobby, or performing some art or music, or meeting with friends, we find such experiences meaningful. The meaning comes to us, phenomenologically speaking, as a kind of inner experience. If it is a more complex situation in our life that we are finding meaning for, then often this is an experience akin to an insight. If our target of insight is reality as such, then what we might find here, after some contemplative effort, is a deep structure of reality. This might be a theoretical structure, the insight about a scientific problem, like Barbara McClintock who found jumping genes that way, or the insight about the relativity of time that Einstein found, etc. Or it might be the insight about aims or values. At the high moral end, it might be the insight about ethical values in general. It seems quite remarkable that different religious and spiritual traditions have some very similar ethical core values, for instance about the sanctity of life, even though some more peripheral values, like the value of property or sexual propriety, might be quite different through ages and cultures. Thus, provided my somewhat essentialist view of values sketched here is at all feasible and at least some core values and ethical principles are part of the deep structure of reality, as I suppose, then it might be possible to discover values as deep structure of reality that way.

In order to make this process a scientific one, the “discoveries” need to become open to discourse and critique. Either they inform theories that can then be clearly communicated, modeled, and empirically tested, or they inform value decisions about research topics and approaches that pragmatically prove their worth after the fact, or they stay in the purely noumenal realm where they become part of a discursive and narrative tradition that interprets and critiques them, as has been the case for theological or religious narratives. But the scientific guidepost is at least open discourse and critique, among others. That there might be more regulative principles that pertain to a future contemplative science is obvious, but since this has to be worked out, it is difficult to be more precise at this point.

## Conclusion: Toward a Contemplative Science

These hints should suffice to make plausible: A different type of ontology, in that case a complementarist ontology that sees consciousness and matter as two complementary aspects of one reality, allows for a different and additional direct access route to reality, more precisely to the deep structure of reality. I call this a contemplative type of science. The way to it would be contemplative, personal practice of scientists. Not everyone would want to or be able to do this, just as not everyone is able to do mathematical modeling or statistical analysis or hermeneutical interpretation. But just as most scientists are grateful that some of their colleagues are able to do statistical analysis or mathematical modeling or hermeneutical interpretation and they know where to turn to should they need help, so it might be useful in future times to have—and welcome—some scientists who are able to access some aspects of reality via this contemplative route, have honed their capacities of intuitive insight and can either help others or can come up with creative ideas, can critique value judgments of others, etc.

Currently, we are nowhere near such a culture and those ideas sound outlandish. But it might well be the case that it might become an asset of scientists that is sought after, just as the capacity of speaking other languages, or being able to program computers, or having learned educational and presentational skills have become assets in recent years that might not have been important a generation ago.

The precise working out where such an epistemology might lead us is in fact quite another and additional enterprise, and the concretization is a project for centuries to come. After all, the working out of our current scientific model has taken more than 500 years, and it is far from satisfying. My purpose here was much more modest: I wanted to show that a broadened ontology would lead to a broadened epistemology. I gave some arguments and reasons why I think we need a broader ontology. And I showed that and how this would lead to a broader epistemology. This allowed us to see some rough outlines of what this would mean. The quest is now open to spell out what such a contemplative science might be able to achieve, where it might be useful and where not, whether to foster it or not, and if so how and what problems it might create and where we would need to be careful. I wager that it might be useful to at least try this avenue, for our current science has very few mechanisms and methods in place other than generating knowledge to help our societies in the current crises, part of which are the result of our current mode of science, after all.

## Data Availability Statement

The original contributions presented in the study are included in the article/supplementary materials, further inquiries can be directed to the corresponding author.

## Author Contributions

The author confirms being the sole contributor of this work and has approved it for publication.

## Conflict of Interest

The authors declare that the research was conducted in the absence of any commercial or financial relationships that could be construed as a potential conflict of interest.

## Footnotes

1 First experiments with optical instruments were already conducted by Robert Grosseteste around 1220 and probably also by Adelard von Bath some generations earlier, but it was Roger Bacon in his optical treatises who codified them.

2 Pars Sexta, De Scientia Experimentalis, Vol. 2, Cap. 1, p. 168f: “Sed duplex est experientia; una est per sensus exteriores. sed haec experientia non-sufficit homini, quia non plene certificat de corporalibus propter sui difficultatem, et de spiritualibus nihil attingit. Ergo oportet quod intellectus hominis aliter juvetur, et ideo sancti patriarchae et prophetae, quo primo dederunt scientias mundo, receperunt illuminationes interiores et non solum stabant in sensu. Et sunt septem gradus hujus scientiae interioris, unus per illuminationes pure scientiales. Alius gradus consistit in virtutibus… p. 171 Septimus consistit in raptibus, et modis eorum secundum quod diversi diversimode capiuntur, ut videant multa, quae non-licet homini loqui. Et qui in his experientiis vel in pluribus eorum est diligenter exercitatus, ipse potest certificare se non solum de spiritualibus, sed omnibus scientiis humanis. necessaria est nobis scientia, quae experimentalis vocatur. Et volo eam explanare, non solum ut utilis est philosphiae, sed sapientiae Dei, et totius mundi regimini—Experience comes in two strands: one through sense experience… but this does not suffice us humans, for it does not give us certainty about material things because of its difficulty, and about spiritual matters it does not attain anything. That is the reason why the human intellect needs further help. And in that way the holy partriarchs and prophets, who have first given knowledge to the world, have received inner illuminations, and have not only remained in the senses… There are seven grades of this inner science, one through the scientific illuminations, another consists in virtues… The seventh consists in extraordinary types of enlightenment according to which different people understand different things such that they can see a lot which humans are not allowed to speak of. And who is well versed in these experiences or in many of them, he can certify himself and others not only about spiritual matters, but about all human sciences… We have need of this science that is called science of experience. Translation HW.

3 One might point to the enormous power of modern artificial intelligence (AI), which, using strategies such as deep learning in massively parallel connected networks, has been able to achieve huge breakthroughs, such as beating chessmasters and masters in Go, even excelling in parlor games such as nominating facts or sentences that refer to a question asked. However, this is overlooking the fact that all deep learning devices depend on the presence of the information in some form on the internet or in rules which can then be harvested for connections by suitable algorithms. Abduction, however, and genuine creativity for that matter, is the introduction of something completely new, which has not been around before. And hence deep learning algorithms, powerful as they may be, cannot be used as templates for the human mind for the time being, and likely for some time to come.

## References

[B1] Abele A. E. Wojciszke B. (2014). “Communal and agentic content in social cognition : a dual perspective model,” in *Advances in Experimental and Social P sychology*, eds Zanna M. P. Olson J. M. (Burlington: Academic Press), 195–255.

[B2] Agazzi E. (ed.) (1991). *The Problem of Reductionism in Science.* Dordrecht: Kluwer.

[B3] Akhilananda S. (1960). *Hindu Psychology: Its Meaning for the West. With an Introduction by Gordon W. Allport.* London: Routledge.

[B4] Alcock J. E. (2003). Give the null hypothesis a chance: reasons to remain doubtful about the existence of PSI. *J. Conscious. Stud.* 10 29–50.

[B5] Aristoteles (1990). *Lehre vom Beweis oder Zweite Analytik (Organon IV).* Hamburg: Meiner.

[B6] Armstrong D. M. (1968). *A Materialist Theory of the Mind.* New York, NY: The Humanities Press.

[B7] Atmanspacher H. Primas H. (2006). Pauli’s ideas on mind and matter int he context of contemporary science. *J. Conscious. Stud.* 13 5–50.

[B8] Bacon F. (1990). *Neues Organon. Lateinisch-deutsch*. *Hrsg. und mit einer Einleitung von. W. Krohn.* Hamburg: Meiner.

[B9] Bacon R. (1859). “Opus tertium, Opus minus, Compendium philosophiae,” in *Opera quaedam hactenus inedita*, Vol. 1 ed. Brewer J. S. (London: Longman & Roberts).

[B10] Bacon R. (1983). *Roger Bacon’s Philosophy of Nature. A Critical Edition, with English Translation, Introduction, and Notes, of De multiplicatione specierum and De Speculis comburentibus.* Oxford: Clarendon Press.

[B11] Bacon R. (1267/1897). *The Opus Majus of Roger Bacon*, Vol. 2 Oxford: Clarendon Press.

[B12] Bankart C. P. Dockett K. H. Dudley-Grant G. R. (2003). “On the path of the Buddha: a psychologist’s guide to the history of Buddhism,” in *Psychology and Buddhism: From Individual to Global Community*, eds Docket K. H. Dudley-Grant G. R. Bankart C. P. (Dordrecht: Kluwer Academic), 13–44.

[B13] Baruss I. Mossbridge J. (2017). *Transcendent Mind: Rethinking the Science of Consciousness.* Washington, DC: American Pychological Association.

[B14] Beauregard M. (2014). The primordial psyche. *J. Conscious. Stud.* 21 132–157.

[B15] Beckermann A. (1989). “Aristoteles, descartes und die beziehungen zwischen philosophischer psychologie und KI-forschung,” in *Gehirn und Bewsusstsein*, ed. Pöppel E. (Weinheim: VCH Verlagsgesellschaft), 105–123.

[B16] Bem D. J. Tressoldi P. Rabeyron T. Duggan M. (2015). Feeling the future: a meta-analysis of 90 experiments on the anomalous anticipation of random future events. *F1000 Res.* 4:1188. 10.12688/f1000research.7177.2 26834996 PMC4706048

[B17] Berkovich-Ohana A. Dor-Ziderman Y. Glicksohn J. Goldstein A. (2013). Alterations in the sense of time, space, and body in the mindfulness-trained brain: a neurophenomenologically-guided MEG study. *Front. Psychol.* 4:912. 10.3389/fpsyg.2013.00912 24348455 PMC3847819

[B18] Bieri P. (1995). “Why is consciousness puzzling?” in *Conscious Experience*, ed. Metzinger T. (Thoverton: Imprint Academic), 45–60.

[B19] Binder T. (2019). *Franz Brentano und sein Philosophischer Nachlass.* Berlin: De Gruyter.

[B20] Bitbol M. Petitmengin C. (2013). A defense of introspection from within. *Constr. Found.* 8 269–279.

[B21] Bohr N. (1966). *Causality and Complementarity: Essays 1958–1962 on Atomic Physics and Human Knowledge.* New York, NY: Vintage.

[B22] Braude S. E. (1987). Psi and our picture of the world. *Inquiry* 30 277–294. 10.1080/00201748708602124

[B23] Brian D. (1996). *Einstein – A Life.* New York, NY: Wiley.

[B24] Brody N. Oppenheim P. (1969). Application of Bohr’s principle of complementarity to the mind-body problem. *J. Philos.* 66 97–113.

[B25] Burtt E. A. (1932). *The Metaphysical Foundations of Modern Physical Science: A Historical and Critical Essay.* London: Routledge and Kegan Paul.

[B26] Cardeña E. (2014). A call for an open, informed study of all aspects of consciousness. *Front. Hum. Neurosci.* 8:17. 10.3389/fnhum.2014.00017 24478682 PMC3902298

[B27] Cardeña E. (2015). The unbearable fear of PSI: on scientific suppression in the 21st century. *J. Sci. Explor.* 29 601–620.

[B28] Cardeña E. (2018). The experimental evidence for parapsychological phenomena: a review. *Am. Psychol.* 73 663–677. 10.1037/amp0000236 29792448

[B29] Carr B. J. (2015). “Higher dimensions of space and time and their implications for psi,” in *Extrasensory Perception: Support, Skepticim and Science*, Vol. 2 eds May E. Marwaha S. (Westport, CT: Greenwood Publishing), 21–61.

[B30] Chalmers D. J. (1996). *The Conscious Mind. In Search of a Fundamental Theory.* Oxford: Oxford University Press.

[B31] Chalmers D. J. (2010). *The Character of Consciousness.* Oxford: Oxford University Press.

[B32] Churchland P. M. (1988). *Matter and Consciousness : A Contemporary Introduction to the Philosophy of Mind.* Cambridge, MA: MIT Press.

[B33] Churchland P. S. (1986). *Neurophilosophy. Toward a Unified Science of the Mind-Brain.* Cambridge, MA: MIT Press.

[B34] Collins H. (2018). *Artifictional Intelligence: Against Humanity’s Surrender to Computers.* Cambridge: Polity Press.

[B35] Comfort N. C. (2001). *The Tangled Field. Barabara McClintock’s Search for the Patterns of Genetic Control.* Cambridge MA: Harvard University Press.

[B36] Costa de Beauregard O. (1998). The paranormal is not excluded from physics. *J. Sci. Explor.* 12 315–320.

[B37] Crombie A. C. (1953). *Robert Grosseteste and the Origins of Experimental Science 1100-1700.* Oxford: Clarendon.

[B38] Dennett D. C. (1991). *Consciousess Explained.* Boston, MA: Little, Brown & Co.

[B39] Descartes R. (1954). *Philosophical Writings.* London: Nelson.

[B40] Descartes R. (1664/2003). *Treatise of Man (Traité de l’Homme)*, ed. (trans.) Hall T. S.) (Amherst, NY: Prometheus Books).

[B41] Dockett K. H. North-Schulte D. (2003). “Transcending self and other: Mahayana principles of integration,” in *Psychology and Buddhism: From Individual to Global Community*, eds Docket K. H. Dudley-Grant G. R. Bankart C. P. (Dordrecht: Kluwer Academic), 215–238. 10.1007/0-306-47937-0_11

[B42] Dor-Ziderman Y. Berkovich-Ohana A. Glicksohn J. Goldstein A. (2013). Mindfulness-induced selflessness: a MEG neurophenomenological study. *Front. Hum. Neurosci.* 7:582. 10.3389/fnhum.2013.00582 24068990 PMC3781350

[B43] Duggan M. Tressoldi P. (2018). Predictive physiological anticipatory activity preceding seemingly unpredictable stimuli: an update of Mossbridge et al.’s meta-analysis. *F1000Res.* 7:407. 10.12688/f1000research.14330.2 30228876 PMC6124390

[B44] Dupré J. Nicholson D. J. (2018). “Towards a processual philosophy of biology,” in *A Manifesto for a Processual Philosophy of Biology*, ed. Nicholson D. J. (Oxford: Oxford University Press), 10.1093/oso/9780198779636.9780198779001.9780198770001

[B45] Dupré L. (2004). *The Enlightenment and the Intellectual Foundations of Modern Culture.* New Haven, CT: Yale University Press.

[B46] Epperson M. (2009). Relational realism: the evolution of ontology to praxiology in the philosophy of nature. *World Futures* 65 19–41. 10.1080/02604020802557367

[B47] Fahrenberg J. (1992). “Komplementarität in der psychophysiologischen Forschung. Grundsätze und Forschungspraxis,” in *Widersprüchliche Wirklichkeit. Neues Denken in Wissenschaft und Alltag: Komplementarität und Dialogik*, eds Fischer E. P. Herzka H. S. Reich K. H. (München: Piper), 43–77.

[B48] Fann K. T. (1970). *Peirce’s Theory of Abduction.* The Hague: Martinus Nijhoff.

[B49] Ferrer J. N. (2013). *Faith in Ayahuasca: An Interview with Shipibo Shaman Guillermo Arévalo.* Available at: www.Sacredhoop.org (accessed April 9, 2020).

[B50] Ferrer J. N. (2018). *Participation and the Mystery.* Albany, NY: State University of New York Press.

[B51] Fischer K. (2015). *Galileo Galieo. Biographie seines Denkens.* Stuttgart: Kohlhammer.

[B52] Foucault M. (1974/1991). *Die Ordnung des Diskurses. Inauguralvorlesung am Collège de France, 2. Dezember 1970.* Frankfurt: Fischer.

[B53] Frankl V. E. (1964). *Man’s Search for Meaning: An Introduction to Logotherapy.* London: Hodder & Stoughton.

[B54] Fulton P. R. (2008). “Anatta: self, non-self, and the therapist,” in *Mindfulness and the Therapeutic Relationship*, eds Hick S. F. Bien T. (London: Guilford Press), 55–71.

[B55] Green T. J. (2016). *Religion for a Secular Age: Max Müller, Swami Vivekananda, and Vedanta.* London: Routledge.

[B56] Grosseteste R. (1981). *Commentarius in Posteriorum Analyticorum Libros.* Florenz: Leo S. Olschki.

[B57] Hakuin. (1994). *The Essential Teachings of Zen Master Hakuin.* Boston, MA: Shambala.

[B58] Hands J. (2015). *Cosmo Sapiens. Human Evolution from the Origin of the Universe.* London: Duckworth.

[B59] Hanson N. R. (1969/2018). *Perception and Discovery: An Introduction to Scientific Inquiry*, 2nd Edn Cham: Springer.

[B60] Hardy G. H. (1937). The Indian Mathematician Ramanujan. *Am. Math. Mon.* 44 137–155. 10.1080/00029890.1937.11987940

[B61] Hasler F. (2015). *Neuromythologie: eine Streitschrift gegen die Deutungsmacht der Hirnforschung.* Bielefeld: Transcript Verlag.

[B62] Heidegger M. (1967). *Wegmarken.* Frankfurt: Klostermann.

[B63] Heinen G. (2012). *Selbst-Handeln bei Epilepsie: Eine Subjektwissenschaftliche Grundlegung einer Psychosomatischen EPILEPTOLOGIE.* Lengerich: Pabst Scientific Publishers.

[B64] Hoche H.-U. (2008). *Anthropological Complementarism. Linguistic, Logical, and Phenomenological Studies in Support of a Third Way Beyond Dualism and Monism.* Paderborn: Mentis Verlag.

[B65] Husserl E. (1909/1977). *Die Krisis der europäischen Wissenschaften und die transzendentale Philosophie [The Crisis of the European Sciences and Transcendental Philosophy].* Hamburg: Meiner.

[B66] Husserl E. (1930/2009). *Ideen zu einer reinen Phänomenologie und phänomenologischen Philosophie.* Humburg: Meiner.

[B67] Ioannidis J. P. A. (2005). Why most published research findings are false. *PLoS Med.* 2:e124. 10.1371/journal.pmed.0020124 16060722 PMC1182327

[B68] Jackson F. (1986). What Mary didn’t know. *J. Philos.* 83 291–295. 16422045

[B69] James W. (1896). Address of the president before the society for psychical research. *Science* 3 881–888. 10.1126/science.3.77.881 17835015

[B70] Jo H.-G. Hinterberger T. Wittmann M. Lhündrup Borghardt T. Schmidt S. (2013). Spontaneous EEG fluctuations determine the readiness potential: Is preconscious brain activation a preparation process to move? *Exp. Brain Res.* 231 495–500. 10.1007/s00221-013-3713-z 24105593

[B71] Jo H.-G. Hinterberger T. Wittmann M. Schmidt S. (2015). Do meditators have higher awareness of their intentions to act? *Cortex* 65 149–158. 10.1016/j.cortex.2014.12.015 25706808

[B72] Jo H.-G. Wittmann M. Borghard T. L. Hinterberger T. Schmidt S. (2014). First-person approaches in neuroscience of consciousness: brain dynamics correlate with the intention to act. *Conscoius. Cogn.* 26 105–116. 10.1016/j.concog.2014.03.004 24705181

[B73] Jonas H. (1980). “Parallelism and compelementarity: the psychophysical problem in Spinoza and in the succession of Niels Bohr,” in *The Philosophy of B. Spinoza*, ed. Kennington R. (Washington, DC: The Catholic University of America Press), 121–130. 10.2307/j.ctv176hf.11

[B74] Jordan P. (1947). *Verdrängung und Komplementarität. Eine philosophische Untersuchung.* Hamburg: Stromverlag.

[B75] Kanigel R. (1991). *The Man who Knew Infinity: A Life od the Genius Ramanujan.* New York, NY: Charles Scribner.

[B76] Keller E. F. (1983/2003). *A Feeling for the Organism. The Life and Work of Barbara McClintock.* New York, NY: W.H Freeman & Co.

[B77] Kelly E. F. Crabtree A. Marshall P. (2015). *Beyond Physicalism: Toward Reconciliation of Science and Spirituality.* Lanham, MD: Rowman & Littlefield.

[B78] Kelly E. F. Kelly E. M. Crabtree A. Gauld A. Grosso M. Greyson B. (2007). *Irreducible Mind. Toward a Psychology for the 21st Century.* Lanham MD: Rowman & Littlefield.

[B79] King M. (2014). The challenge of research into religion and spirituality. *J. Study Spiritual.* 4 106–120. 10.1179/2044024314z.00000000026

[B80] Krippner S. Sulla J. (2000). Identifying spiritual content in reports from Ayahuasca sessions. *Int. J. Transpers. Stud.* 19 59–76. 10.24972/ijts.2000.19.1.59

[B81] Leibniz G. W. (1966). “Monadologie (1714),” in *Hauptschriften zur Grundlegung der Philosophie*, eds Buchenau A. Cassirer E. (Hamburg: Meiner), 435–456.

[B82] Lindberg D. C. (1992). *The Beginnings of Western Science: The European Scientific Tradition in Philosophical, Religious, and Institutional Context, 600 BC to AD 1450.* Chicago, IL: University of Chicago Press.

[B83] Lindberg D. C. (1997). “Roger Bacon on light, vision, and the universal emanation of force,” in *Roger Bacon and the Sciences*, ed. Hackett J. (Leiden: Brill), 243–275.

[B84] Lucadou W. V. Römer H. Walach H. (2007). Synchronistic phenomena as entanglement correlations in generalized quantum theory. *J. Conscious. Stud.* 14 50–74.

[B85] Lutz A. Jha A. P. Dunne J. D. Saron C. D. (2015). Investigating the phenomenological matrix of mindfulness-related practices from a neurocognitive perspective. *Am. Psychol.* 70 632–658. 10.1037/a0039585 26436313 PMC4608430

[B86] MacPhail J. (2013). *Learning in Depth: A Case Study in Twin 5×5 Matrices of Consciousness.* Ph.D. thesis, Europa-Universität Viadrina, Frankfurt.

[B87] MacPhail J. (2017). Vertical hierarchy and the invariance principle in four models of consciousness/spirituality. *J. Study Spiritual.* 7 99–113. 10.1080/20440243.2017.1370906

[B88] Mandonnet P. (1910). Roger Bacon et le Speculum Astonomiae (1277). *Rev. Néoscolastique Philos.* 17 313–335. 10.3406/phlou.1910.2750

[B89] Maxwell N. (1968). Understanding sensations. *Aust. J. Philos.* 46 127–146.

[B90] Maxwell N. (1984). *From Knowledge to Wisdom: A Revolution in the Aims and Methods of Science.* Oxford: Blackwell.

[B91] Maxwell N. (2000). The mind-body problem and explanatory dualism. *Philos. Context* 75 49–71. 10.1017/s003181910000005x

[B92] Maxwell N. (2011). Three philosophical problems about consciousness and their possible resolution. *Open J. Philos.* 1:1 10.4236/ojpp.2011.11001

[B93] Maxwell N. (2017). *In Praise of Natural Philosophy. A Revolution for Thought and Life.* Montreal: McGill-Queen’s University Press.

[B94] May E. C. Marwaha S. B. (eds) (2018). *The Star Gate Archives. Reports of the United States Government Sponsored Psi Program, 1972–1995: Remote Viewing, 1972–1984*, Vol. 1 Jefferson, NC: Mc Farland.

[B95] May E. C. Utts J. M. Trask V. V. Luke W. W. Frivold T. J. Humphrey B. S. (2018). “Review of the psychoenergetic research conducted at SRI International (1973–1988),” in *The Star Gate Archives. Reports of the United States Government Sponsored Psi Program, 1972–1995: Remote Viewing, 1972–1984*, Vol. 1 eds May E. C. Marwala S. B. (Jefferson, NC: McFarland), 495–504.

[B96] Meier C. A. (ed.) (1992). *Wolfgang Pauli und C.G. Jung. Ein Briefwechsel 1932–1958.* Heidelberg: Springer.

[B97] Meier C. A. (ed.) (2001). *Atom and Archetype: The Pauli/Jung Letters 1932–1958.* Princeton, NJ: Princeton University Press.

[B98] Merleau-Ponty M. (1966). *Die Phänomenologie der Wahrnehmung.* Berlin: de Gruyter.

[B99] Meyer-Abich K. M. (1965). *Korrespondenz, Individualität und Komplementarität.* Wiesbaden: Steiner.

[B100] Mossbridge J. A. Tressoldi P. E. Utts J. (2012). Predictive physiological anticipation preceding seemingly unpredictable stimuli: a meta-analysis. *Front. Psychol.* 3:390. 10.3389/fpsyg.2012.00390 23109927 PMC3478568

[B101] Nagel T. (1974). What is it like to be a bat? *Philos. Rev.* 83 435–450.

[B102] Nagel T. (2012). *Mind and Cosmos: Why the Materialist Neo-Darwinian Conception of Nature is almost Certainly False.* Oxford: Oxford University Press.

[B103] Newbold W. R. (1921). The Voynich Roger Bacon manuscript. *Trans. Coll. Phys.* 43 431–474.

[B104] Noë A. (2009). *Out of Our Heads: Why You are Not your Brain, and Other Lessons from the Biology of Consciousness.* New York, NY: Hill & Wang.

[B105] Open Science Collaboration (2015). Estimating the reproducibility of psychological science. *Science* 349:aac4716. 10.1126/science.aac4716 26315443

[B106] Peirce C. S. (1931). *Collected Papers.* Cambridge, MA: Harvard University Press.

[B107] Penrose R. (1990). Précis of The Emperors New Mind: concerning computers, minds, and the laws of physics. *Behav. Brain Sci.* 13 643–705. 18240751

[B108] Petitmengin C. (2006). Describing one’s subjective experience in the second person. An interview method for the science of consciousness. *Phenomenol. Cogn. Sci.* 5 229–269. 10.1007/s11097-006-9022-2

[B109] Petitmengin C. (2007). Towards the source of thought: the gestural and transmodel dimension of lived experience. *J. Conscious. Stud.* 14 54–82.

[B110] Petitmengin C. Bitbol M. (2009). The validity of first-person descriptions as authenticity and coherence. *J. Conscious. Stud.* 16 363–404.

[B111] Petitmengin C. Navarro V. Le Van Quyen M. (2007). Anticipating seizure: pre-reflective experience at the center of neuro-phenomenology. *Conscious. Cogn.* 16 746–764. 10.1016/j.concog.2007.05.006 17590351

[B112] Plato (1967). *Briefe, Griechisch-Deutsch.* München: Ernst Heimeran.

[B113] Power A. (2012). *Roger Bacon and the Defence of Christendom.* Cambridge: Cambride University Press.

[B114] Primas H. (1981). *Chemistry, Quantum Mechanics and Reductionism.* Heidelberg: Springer.

[B115] Primas H. (1991). “Reductionism: Palaver without precedent,” in *The Problem of Reductionism in Science*, ed. Agazzi E. (Dordrecht: Kluwer), 161–172. 10.1007/978-94-011-3492-7_9

[B116] Primas H. (1994a). “Endo- and exo-theories of matter,” in *Inside versus Outside*, eds Atmanspacher H. Dalenoort G. J. (Heidelberg: Springer), 163–193. 10.1007/978-3-642-48647-0_10

[B117] Primas H. (1994b). “Realism and quantum mechanics,” in *Proceedings of the Ninth International Congress of Logic, Methodology and Philosophy of Science*, Upsala, 609–631.

[B118] Primas H. (1996). “Time-asymmetric phenomena in biology. Complementary exophysical descriptionsarising from deterministic quantum endophysics,” in *Proceedings of the International Workshop on Information Biothermodynamics*, Torn.

[B119] Primas H. (1997). “The representation of facts in physical theories,” in *Time, Temporality, Now. Experiencing Time and Concepts of Time in an Interdisciplinary Perspective*, eds Atmanspacher H. Ruhnau E. (Berlin: Springer), 243–265. 10.1007/978-3-642-60707-3_18

[B120] Principe L. (2016). “Scientism and the religion of science,” in *Scientism: The New Orthodoxy*, eds Williams R. N. Robinson D. N. (London: Bloomsbury), 41–61.

[B121] Radin D. (2018). *Real Magic. Ancient Wisdom, Modern Science, And A Guide to the Secret Power of the Universe.* New York, NY: Harmony Books.

[B122] Rao K. R. (2005). Perception, cognition and consciousness in classical Hindu psychology. *J. Conscious. Stud.* 12 3–30.

[B123] Reber A. S. Alcock J. E. (2020). Searching for the impossible: parapsychology’s elusive quest. *Am. Psychol.* 75 391–398. 10.1037/amp0000486 31192620

[B124] Robinson D. N. (2016). “Science, scientism, and explanation,” in *Scientism: The New Orthodoxy*, eds Williams R. N. Robinson D. N. (London: Bloosmbury), 23–40.

[B125] Röhrle E. A. (2001). *Komplementarität und Erkenntnis: Von der Physik zur Philosophie.* Münster: Lit-Verlag.

[B126] Römer H. (2012). Why do we see a classical world? *Trav. Math.* 20 167–186.

[B127] Römer H. Walach H. (2011). “Complementarity of phenomenal and physiological observables: a primer on generalised quantum theory and its scope for neuroscience and consciousness studies,” in *Neuroscience, Consciousness and Spirituality*, eds Walach H. Schmidt S. Jonas W. B. (Dordrecht: Springer), 97–107. 10.1007/978-94-007-2079-4_7

[B128] Schmidt S. (2012). Can we help just by good intentions? A meta-analysis of experiments on distant intention effects. *J. Altern. Complement. Med.* 18 529–533. 10.1089/acm.2011.0321 22784339

[B129] Schmidt S. Schneider R. Utts J. Walach H. (2004). Remote intention on electrodermal activity - Two meta-analyses. *Br. J. Psychol.* 95 235–247. 15142304 10.1348/000712604773952449

[B130] Schwartz G. E. Woollacott M. Schwartz S. Barušs I. Beauregard M. Dossey L. (2018). The academy for the advancement of postmaterialist sciences: integrating consciousness into mainstream science. *J. Sci. Heal.* 14 111–113. 10.1016/j.explore.2017.12.006 29475815

[B131] Searle J. R. (1992). *The Rediscovery of the Mind.* Cambridge, MA: Massachusetts Institute of Technology Press.

[B132] Sedlmeier P. Kunchapudi S. (2016). How do theories of cognition and consciousness in ancient Indian thought systems relate to current Western theorizing and research? *Front. Psychol.* 7:343. 10.3389/fpsyg.2016.00343 27014150 PMC4791389

[B133] Shanon B. (2002). Ayahuasca visualizations: a structured typology. *J. Conscious. Stud.* 9 3–30.

[B134] Shear J. (2007). “Eastern methods for investigating mind and consciousness,” in *The Blackwell Companion to Consciousness*, eds Velmans M. Schneider S. (Oxford: Blackwell), 670–710.

[B135] Spinoza B. D. (1977). *Die Ethik. Lateinisch und Deutsch.* Stuttgart: Reclam Verlag.

[B136] Storm L. Sherwood S. J. Roe C. A. Tressoldi P. E. Rock A. J. Di Risio L. (2017). On the correspondence between dream content and target material under laboratory conditions: a meta-analysis of dream ESP studies, 1966–2016. *Int. J. Dream Res.* 10 120–140.

[B137] Storm L. Tressoldi P. (2017). Gathering in more sheep and goats: a meta-analysis of foreced-choice sheep-goat ESP studies, 1994–2015. *J. Soc. Psych. Res.* 81 79–107.

[B138] Storm L. Tressoldi P. Utts J. (2013). Testing the Storm et al. (2010) meta-analysis using Bayesian and frequentist approaches: Reply to Rouder et al. (2013). *Psychol. Bull.* 139 248–254. 10.1037/a0029506 23294093

[B139] Storm L. Tressoldi P. E. Di Risio L. (2010). Meta-analysis of free-response studies, 1992–2008: assessing the noise reduction model in parapsychology. *Psychol. Bull.* 136 471–485. 10.1037/a0019457 20565164

[B140] Storm L. Tressoldi P. E. Di Riso L. (2012). Meta-analysis of ESP studies, 1987–2010: assessing the success of the forced choice design in parapsychology. *J. Parapsychol.* 76 243–273.

[B141] Suzuki S. (1970). *Zen Mind, Beginners Mind.* New York, NY: Weatherhill.

[B142] Targ R. Puthoff H. (1974). Information transfer under conditions of sensory shielding. *Nature* 251 602–607. 10.1038/251602a0 4423858

[B143] Targ R. Puthoff H. E. May E. C. (1979). “Direct perception of remote geographic locations,” in *Mind at Large*, eds Tart C. T. Puthoff H. E. Targ R. (New York, NY: Praeger), 78–106.

[B144] Thomas Gallus (1243/1503^∗^). *De Septem Gradibus. Incipit Tractatus (ut fertur) Sancti Bonaventurae de Septem Gradibus Contemplationis.* (Paris: Gaspard Phillipe).

[B145] Tiefensee E. (1998). *Philosophie und Religion bei Franz Brentano (1838–1917).* Tübingen: Francke.

[B146] van Fraassen B. (2002). *The Empirical Stance.* New Haven, CT: Yale UP.

[B147] van Fraassen B. (2016). “Naturalism in epistemology,” in *Scientism: The New Orthodoxy*, eds Williams R. N. Robinson D. N. (London: Bloomsbury), 64–95.

[B148] Varela F. (1981a). “Autonomy and autopoiesis,” in *Self-Organizing Systems*, eds Roth G. Schwengler H. (Frankfurt: Campus Verlag), 14–23.

[B149] Varela F. J. (1981b). “Describing the logic of the living. The adequacy and limitation of the idea of autopoiesis,” in *Autopoiesis: A Theory of Living Organization*, ed. Zeleny M. (New York, NY: Elsevier), 36–48.

[B150] Varela F. J. (1984). “The creative circle: Sketches on the natural history of circularity,” in *The Invented Reality. How Do We Know What We Believe We Know?: Contributions to Constructivism*, ed. Watzlawick P. (New York, NY: Norton), 309–324.

[B151] Varela F. J. (1996). Neurophenomenology: a methodological remedy for the hard problem. *J. Conscious. Stud.* 3 330–349.

[B152] Varela F. J. Thompson E. Rosch E. (1991). *The Embodied Mind. Cognitive Science and Human Experience.* Cambridge, MA: MIT Press.

[B153] Velmans M. (2007). “An epistemology for the study of consciousness,” in *The Blackwell Companion to Consciousness*, eds Velmans M. Schneider S. (New York, NY: Blackwell), 711–725. 10.1002/9780470751466.ch56

[B154] Velmans M. (2009). *Understanding Consiousness.* London: Routledge.

[B155] Velmans M. (1993/2007). “A Reflexive Science of Consciousness,” in *Experimental and Theoretical Studies of Consciousness*, eds Bock G. R. Marsh J. (Chichester: Wiley), 81–99. 10.1002/9780470514412.ch5 8319514

[B156] Walach H. (2005). The complementarity model of brain-body relationship. *Med. Hypotheses* 65 380–388. 10.1016/j.mehy.2005.01.029 15922117

[B157] Walach H. (2009). A medieval Carthusian monk’s recipe to multiple kensho: Hugh of Balma’s approach to mystical union and some striking similarities to modern Zen teaching. *Stud. Spiritual.* 19 199–225. 10.2143/sis.19.0.2043680

[B158] Walach H. (2010). *Notitia Experimentalis Dei – Experiential Knowledge of God: Hugh of Balma’s Mystical Epistemology of Inner Experience – A Hermeneutic Reconstruction.* Salzburg: Institut für Anglistik.

[B159] Walach H. (2015). *Secular Spirituality: The Next Step Towards Enlightenment.* Dordrecht: Springer.

[B160] Walach H. (2017). Secular spirituality–What it is. Why we need it. How to proceed. *J. Study Spiritual.* 7 7–20. 10.1080/20440243.2017.1290028

[B161] Walach H. (2019). *Beyond a Materialist Worldview: Towards an Expanded Science.* London: Scientific and Medical Network.

[B162] Walach H. Ludacou W. V. Römer H. (2014). Parapsychological phenomena as examples of generalized non-local correlations – A theoretical framework. *J. Sci. Explor.* 28 605–631.

[B163] Walach H. Römer H. (2000). Complementarity is a useful concept for consciousness studies. A reminder. *Neuroendocrinol. Lett.* 21 221–232. 11455354

[B164] Walach H. Römer H. (2011). “Generalized entanglement – A nonreductive option for a phenomenologically dualist and ontologically monist view of consciousness,” in *Neuroscience, Consciousness and Spirituality*, eds Walach H. Schmidt S. Jonas W. B. (Dordrecht: Springer), 81–95. 10.1007/978-94-007-2079-4_6

[B165] Walach H. Runehov A. L. C. (2010). The epistemological status of transpersonal psychology: The data-base argument revisited. *J. Conscious. Stud.* 17 145–165.

[B166] Walach H. Schmidt S. (2005). Repairing Plato’s life boat with Ockham’s razor: the important function of research in anomalies for mainstream science. *J. Conscious. Stud.* 12 52–70.

[B167] Walach H. Tressoldi P. Pederzoli L. (2015). *Mental, Behavioural and Phyisiological Nonlocal Correlations within the Generalized Quantum Theory Framework: A Review.* Available online at: https://ssrn.com/abstract=2695741 (accessed April 9, 2020).

[B168] Weger U. Wagemann J. (2015). The behavioral, experiential and conceptual dimensions of psychological phenomena: body, soul and spirit. *New Ideas Psychol.* 39 23–33. 10.1016/j.newideapsych.2015.07.002

[B169] Williams R. N. Robinson D. N. (eds) (2016). *Scientism: The New Orthodoxy.* London: Bloomsbury.

[B170] Winter U. Levan P. Borghardt T. L. Akin B. Wittmann M. Leyens Y. (2020). Content-free awareness: EEG-fcMRI correlates of consciousness as such in an expert meditator. *Front. Psychol.* 10:3064. 10.3389/fpsyg.2019.03064 32132942 PMC7040185

[B171] Zahavi D. (2003). *Husserl’s Phenomenology.* Stanford, CA: Stanford University Press.

